# Bacteria the Cause of Caries

**Published:** 1882-09

**Authors:** F. Y. Clark

**Affiliations:** 35 West Thirty-fifth street; N. Y. City


*FOR CONTENTS OF THIS NUMBER SEE PAGE* 560.

*THE*

### Independent Practitioner.

VOL. III.——*September*, 1882.——NO. 9.

### ORIGINAL COMMUNICATIONS.

*We are not responsible for the opinions expressed by contributors.*

ARTICLE I.

### BACTERIA THE CAUSE OF CARIES.

Read before the American Dental Convention August, 1882.

BY F. Y. CLARK, M. D., D. D. S., N. Y. CITY.

There is much confusion and contradiction as to the cause of dental caries. It has ever been taught in our schools, books, and until very recently by nearly every practitioner of dentistry, to be of acid origin; but the facts gained from analysis and microscopical investigation, to say nothing of overwhelming circumstantial evidence, does not warrant this teaching or belief. Over fifteen years ago we asked, in an essay before the Southern States Dental Association, What is carious dentine? How does it differ from sound tooth structure? Is it the same when detached from as when connected with sound tooth structure? Will it, when taken from its bed and placed under a filling, act on the living the same as when allowed to remain partially connected with living structure? Why are we so particular in the removal of every mite before filling? You say because, if any is allowed to remain, in the course of time it will infect sound dentine, and necessitate refilling. Would a layer of sound dentine, or any less objectionable substance, do this? You answer no. What contagious agent, then, is there in this mite of decay, to produce this? How does the same agent produce all the different shades of decay—the black, the white, the yellow, and so on? We are told acetic fermentation and acids combining with the lime of the tooth form new agents; but what those agents are, or how they act on sound structure, we are not intelligently informed.

Our fermentations, as far as we have any understanding, are alcoholic, acetic, butric or lactic, ammoniacal, and putrefactive. Fermentation is the converting of organic or inorganic matter into something else. There is no fermentation without an organism. It is through air germs that first ferment is introduced, and the organisms inaugurated by these first germs inaugurate a second, and so on, to putrefaction. It is through this law of succession of organic life that all vegetation is produced. Without it earth would be a rocky waste, with no vegetation or means of sustaining animal life. It is through air germs that the first ferment of which we have any knowledge is brought about, and to the organisms thus introduced investigation as to the laws governing higher life must begin. Exclude air from any organic matter, and it will remain unacted on and unchanged indefinitely. This is seen in every day life, for there is no animal or vegetable matter about us that is not undergoing change through this law. Whenever any production of the vegetable kingdom from want of suitable sustenance, or the conditions necessary to a healthy development, becomes wanting or deficient, it is quickly attacked by fungi, or organisms which increase in many instances so rapidly as to destroy adjacent healthy growths. This is sometimes the source of great loss and annoyance to agriculturists, as frequently whole fields are destroyed. Now the same thing occurs in the animal kingdom when there is a want of bioplasm, necessary for the building up of a member of any organism. In this bioplasm is cell matter that constitutes enamel, dentine, vein, artery, and every tissue of life, and when wanting or interrupted, no matter how caused, ferment through air germs follow, and thus are inaugurated and brought to view by the microscope the smallest living organisms called bacteria. When through the microscope we see for the first time these myriads of living, moving organisms, apparently endowed with some kind of intelligence and movement power, which enables them to swim about, turn, twist, go backward and forward, not more than 1–3,000 of an inch in diameter, all occupying a space not larger than the point of a needle, and are told they are nothing but plants intimately related to larger ones familiar to common vision, no wonder the mind is held in doubt, and is half inclined to disbelieve one of its own senses. But farther examination opens up a new world of investigation. We study a few works, spend a few months with the microscope, and think with Darwin and many others that evolution is a fact; that some one of these infinitesimal specks is certainly within the reach of man's production; but when we get farther on in the paths of Pasture, Huxley, Tyndal, Cohn and Beal, and become somewhat familiar with some of their experiments, we are obliged to own that the misty veil of first life is still unrent. If air germs, as some say, come through dust from some other planet, we may ask how come they in this dust. So, with present powers of research, we must own, as Beal puts it, there is a beyond and beyond, which man cannot reach. Through these smallest of living things decay becomes, as the saying is, but another form of life. When all of man that is earthy ceases countless lives are inaugurated, which in turn, through fermentation, give life to successors, and so on up the organic ladder until man is again living through the agency that brought about his putrefaction, and thus life is a circle. At one time it was a mooted question to what kingdom bacteria belonged. Many investigators classed them with infusoria, and other low organisms of animal life, but now they are generally admitted to be plants. They differ in nearly all respects from infusoria. They have no morphological distinction of ends, no flagellata, or movement organs, and live like the ameeba by absorption—by extracting what is necessary for the propagation of their species from the substance in which they are found, organic or inorganic. They are of almost every conceivable shape—round, oval, twisted, straight, oblong, screw shape, cylindrical, and many others. They live with or without air, and are found in all animal and vegetable matters undergoing fermentation. Sometimes they are very active, and this is lasting when seen in a jelly-like mass; and again, without apparent cause, they become motionless. Sweets and fermentation increase their activity. Through the protection of a cell membrane they are enabled to resist great extremes of heat and cold, some species much more than others. We have frequently seen spirellum vultans—the largest of all bacteria—converted into ice, and become quite lively when thawed out. They are propagated by cell division, or by the formation of new joints, and subsequent separation at these joints. In favorable and unmolested localities, when conditions are suitable, their increase is almost beyond conception. One or two will increase to millions within an hour. It is by budding their species become mixed, as on an apple tree, by budding or grafting we have several varieties of apples—sweet, tart, sour—all different in taste, shape, and shade ; although the wood and bark of tree and limb is the same, the fruit is different. Thus a contagious germ can spring from a harmless parent cell, just as the poison ivy can be budded on the harmless woodbine, or the poison bitter almond on the nutritious sweet. So, also, in the lowest order of plant life, owing to causes as yet not thoroughly understood, contagious germs can spring from harmless parent cells. All tissues, veins, arteries, bones, teeth, and every special part of the body, have particular special and individual cells, all acting in harmony constructing, building up, repairing, throwing off and removing defective and useless parts. If a cell necessary for the perfect development of a tooth is destroyed, or in any way disturbed, the tooth on erupting will invariably show the disturbance by defective enamel pits, fissures, or other marks. Owing to this, we frequently see whole sets on erupting defective. Thus we can readily understand how any organ can become defective, diseased, or missing, if these bioplastic cells are in any way interrupted. It is owing to due respect to this law that certain teeth are sometimes wanting and found in remote parts. As stated, the chemical theory of decay is, that acids generated in the mouth act on tooth structure so as to cause its decay. But with the death of Liebig, and the advancement of microscopy, this chemical school, with its nascent and atomic theories is fast passing away, and it would now be a bold scientist who should dare to deny the crucical evidence of Pasteur "that fermentation is a change of chemical composition in organic compounds brought about by a certain class of plants, which deprived of drawing the essential element of their composition from the air, *propagate themselves in the very substance of organic matters of all kinds by the breaking up of these matters and appropriating such elements as are necessary to their own growth.* Organic matter cannot of itself bring about inorganic matter, no more than inorganic can bring about organic. This in microscopy is an accepted fact, as sterilized fluids will remain so for ages if kept free of external matter, but when exposed to air, if pabulum be added, ferment follows until that of putrefication sets in, and with putrefication comes bacteria termo, an organism ever present where and when putrefaction is going on. Although bacteria termo is seen with other species, it is accepted as the true organism of putrefaction, and no putrefaction can take place without the assistance of this germ, bacteria termo, which has somewhat to do with our inquiry, has been generally plated and noticed, but until recently its true ferment has been little understood. If a piece of meat is placed in a glass of water after other ferments have passed and putrefication set in, a cloud will be seen surrounding the meat, and will increase and continue active as long as there is anything to putrefy—this cloud is bacteria termo. Towards the last the cloudy sediment changes color and becomes very offensive, so much so that it is painful to examine. As before stated, when the teeth are imperfectly formed—full of pits and uneven surfaces, owing to derangement of form cells—they are more easily acted on by external agents. These pits, fissures, and roughened surfaces are, in many cases, inaccessible to the brush, and consequently become little laboratories for fermentation. If a mite of animal or vegetable food is left for a few days in or around the teeth, one ferment follows another, with organism after organism, until the tearing down process of putrefication or bacteria termo is in full force. One grain of vegetable or animal matter, with a few drops of saliva placed in a glass, and kept at the normal temperature of the mouth, will generate within a few days millions of bacteria. The same thing goes on in the mouth whenever or whereever these organisms find pabulum and unmolested localities. This is a universally accepted fact with the belief of the majority, that the cause of decay is owing to acids in the saliva. After alcoholic fermentation it is true we have acetic ; but the acid of this ferment is so weak in the fluids of the mouth that it can scarcely be detected, and in very many mouths where the teeth are under rapid decay it or any other acid is not found at all. A tooth may be cut half away, and if smoothly polished it will never decay. If caries was the result of acetic acid in the saliva, would this be the case ? Mycoderma aceti, the supposed ferment of vinegar, is not perfectly understood. Particles of food in pits and fissures of the teeth, where mycoderma aceti is found, has a slight yellow tint; following other organisms, such as the vibro regula and spirochœte plicatilis, are seen ; but so far as can be seen there is no perceptible action on the tooth structure until bacteria termo is present. As we said in a previous paper, it is impossible to account for or explain all the shades of carious dentine on the acid theory, for acids cannot by any law known to chemistry produce them. But on the other hand, by the pigment in the protoplasm of bacteria, all the shades seen can be produced. We don't wish to advocate the chewing of tobacco, for in the mouth it is a dirty weed, but every dentist of any experience and ordinary observation knows it has a tendency to stop decay, which no doubt is owing to the antiseptic agency of nicotine. The crown of a tooth, as you all know, is protected by a covering of enamel, which is very dense ; under this covering there is what is known as dentine, which is more like bone full of tube cells, with mouths opening into the enamel. These cells, called canacliculi, are filled with nerve bioplasm, and, by measurement, are large enough for the easy entrance of many species of bacteria. Thus, from what we have said, those familiar with the laws of fermentation can begin to understand how this bioplasm—the life of the tooth—may be absorbed or converted into pabulum for distructive organisms. For, as already stated, when and whereever the enamel, through imperfect developent or other cause, is thin, as under the pits or abraded surfaces, the mouths or tubes of the dentine through fermentation are easily entered by bacteria, which, as Pasture and others assert, live at the expense of the organism in which they are found, by absorbing and appropriating the life matter for their own substance and the propagation of their species. We will now attempt to show a few of the organisms found in the saliva of the mouth and carious dentine. The microscope before you has a B ocular and a one-sixteenth objective. Examine first on this slide No. 1 a drop taken from the sub-lingual duct of a gentleman present in whose mouth are several decayed teeth ; you will find in this saliva no organism, at least none that the power used will bring to view. Now examine this slide No. 2, on which is the fraction of another drop from the same mouth, taken with a little mucus from the pit of a second superior molar. In this you see spherical organisms, which we will not attempt at present to name or class. They are in many respects like micrococus fulvus, and are generally found in first ferments. On this slide, No. 3, we have a little saliva taken from my own mouth, which has been kept at normal temperature for nearly two days, and which is now in a state of fermentation. In this, you see, the organisms are larger, quite numerous, and active. They are slightly different in shape, but no doubt of the same species as seen on slide No. 2.

In this vial No. 4 is carious dentine mucus and saliva, which has been kept at the temperature of the mouth for forty-eight hours. In this preparation you see spirochate plicatiles, vibrio regular and what we take to be bacteria termo. Allow this preparation to remain a few days longer, and it will become so putrid and offensive as to scent this whole hall, so that no organism but bacteria termo will live in it. You meet with patients now and then with the accumulation of weeks, in and around their teeth, who never use a brush whose breath is equally offensive ; for where particles of decomposing food remain undisturbed, through fermentation, the same thing goes on. There are one or two other preparations of carious tooth structure that we would like you to examine but fear time will not permit. Enough has been said and seen to create inquiry if not conviction as to organic decay. The time is fast approaching when thinking minds will not be satisfied with the *old egg shell theory* that teeth are nothing but lumps of lime, and that the acetic acid of the mouth decomposes them. This theory, as ridiculous and untenable as it may seem, has misled and placed our profession in a wrong position for over an age, and by false practice caused the loss of many teeth. But when we see in letters and journals such passages as the following from such men as Dr. Spaulding, of St. Louis, and Stockwell, of Springfield, Mass., there is reason to look forward to better teaching and more intelligent practice. In the March Number of the New England Journal, Dr. Spaulding says :

"My first experiments were made upon the saliva of a young girl (aged 12), whose teeth were decaying rapidly, so much so that fillings previously inserted, that is, before the case came into my hands, whether of gold or other materials, had lasted but a short time, and in whose teeth new cavities were constantly forming, and this at so rapid a rate that cavities of considerable size would form in the space of a few months, notwithstanding pretty thorough cleanliness and good general care. I looked to find an acid reaction of the saliva in this case, and had been reflecting on a course of medical treatment, having in view this supposed condition of the saliva. What then was my surprise on finding after repeated tests that the saliva of this young person exhibited in every test either a neutral or a slightly alkaline chemical reaction. In no one of a large number of tests was there any, even the smallest, acid reaction shown. I immediately sought other cases where a similar distinctive process was going on, but the result in each was precisely the same as the case just narrated."

Dr. C. T. Stockwell writes: "The more I look into this thing the more convinced I am that the present and former teachings on the subject of Etiology of dental decay are erroneous and superficial. That acids are *not* the exciting cause. In fact, that acids have very little to do with it. The ever present germ and bacterial life in all stages of developement, together with a *weakened condition* of the nervous tissues, are the two necessary conditions of tooth decay. The laws and processes of *putrefaction* come in here as the '*sub*-structure' upon which we have to erect *our* '*super* structure.' Is this not so? This is a subject that will no longer down at the cold sneer or shrug of the self-sufficient shoulder. The profession have got to meet this question and prove it erroneous or accept it with all an acceptance implies." In conclusion then, in order to be correctly understood, we repeat that acids are not the cause of carious tooth structure. The real cause is bacteria inaugurated by air germs through first ferment. All pits, fissures, abraded, hidden, and all inaccessible places in and around the teeth, where particles of food or foreign matters of any kind is lodged and unmolested, organism follows organism, *absorbing the bioplasm in the canaliculi, leaving the limey substance to disintegrate. The change of shade is caused by the pigment in the protoplasm* of bacteria, which is light, yellow, brown or dark, according to organic advancement. In disinfecting carious structure before filling, where there is danger of pulp exposure, we have been in the habit of using carbolic acid, chloride of zinc, or chloride of mercury, and have had the satisfaction in after years of seeing the good results of this practice, and in quite a number of cases positive recalcification. Dr. R. Koch's experiments on bacilla, shows that chlorine bromine and mercuric chloride gives the best results. Solutions of mercuric chloride nitrate or sulphate, diluted to one in 100, destroy spores in ten minutes in prepared solutions. Does not this solve the mystery of the old mercurial fillings inserted over forty years ago and explain the cause of black recalcification of dentine often seen under those fillings. Amalgam at that time, as you know, was composed of coin silver and mercury, and the men using it were considered quacks and made little or no attempt of removing decay or surplus mercury. Our modern amalgams though *very pure*, and which takes so *very little mercury*, don't give the same results.

35 West Thirty-fifth street.

